# Association of the acute:chronic workload ratio and wellness scores in premier league male hockey players

**DOI:** 10.17159/2078-516X/2021/v33i1a9244

**Published:** 2021-08-11

**Authors:** GN Veiga, G Torres, I Maposa

**Affiliations:** 1 Centre for Exercise Science and Sports Medicine, School of Therapeutic Sciences, Faculty of Health Science, University of the Witwatersrand, Johannesburg, South Africa; 2 Division of Epidemiology and Biostatistics, Faculty of Health Sciences, University of the Witwatersrand, Johannesburg, South Africa

**Keywords:** training workload management, training load, session rating of perceived exertion, fatigue, muscle soreness

## Abstract

**Background:**

The relationship between acute:chronic workload ratios (ACWR) and the incidence of injury, as well as the relationship between subjective wellness scores and training load, is unclear in hockey players.

**Objectives:**

This study investigated these relationships to determine if the ACWR is a useful workload management tool for field hockey players. In addition, the study investigated the association between specific subjective wellness scores (fatigue, sleep quality, general muscle soreness, mood and stress level) and the acute:chronic workload ratio and training load. The study also assessed the association between individual players’ training loads with the self-reported wellness scores.

**Methods:**

Fourteen male field hockey players from the men’s first team at the University of the Witwatersrand participated in the study over ten weeks during a premier league competition phase. A Google form was completed within thirty minutes of every training session/match. This form consisted of questions that allowed for the calculation of the ACWR.

**Results:**

No incidence of injury was reported during the ten-week period. ACWR scores ranged between 0.67 and 1.87. The ACWR was associated with general muscle soreness (p = 0.010) and training load was associated with fatigue (p = 0.002), sleep quality (p = 0.05), general muscle soreness (p = 0.004), and mood (p=0.025).

**Conclusion:**

There may be some merit in the use of subjective wellness measures as workload management tools in field hockey. Further research is required to determine if there is an optimal ACWR for this sport.

The primary objective of sports practitioners is to minimise injuries within a team while simultaneously optimising the players’ training load. Team sports players are required to maintain the high level of fitness developed during the pre-season training period throughout the competition phase. In addition, players need to recover from training sessions and manage fatigue levels.^[1]^ This fragile balance of training and recovery poses a challenge for sports practitioners therefore athlete monitoring systems are required to be in place. While monitoring workload through the use of objective measures (such as, GPS data of distance covered or heart rate) has been extensively used for the workload management of the athlete, subjective measures (such as, session rating of perceived exertion (sRPE) and wellness scores (e.g. muscle soreness, fatigue, sleep quality) have only just begun to gain momentum in the world of sports science. The reliability and validity of such measures have been well-documented.^[2]^ The advantage of utilising subjective measures is that they equate to the athlete’s internal load which is specific to each individual athlete. This link between subjective measures and internal load is of importance in training because it tells us how well the athlete is adapting to the training load imposed.^[3]^

The acute:chronic workload ratio (ACWR) has been studied extensively over multiple sports disciplines. Studies investigating the viability of ACWR as a workload management tool have been conducted with Australian rules football players^[4]^, rugby players^[5]^, soccer players^[6]^ and Gaelic football players^[7]^, but there is a lack of these types of studies for field hockey players. Hockey is unique when compared to other sports as it uses rolling substitutions during gameplay. This could affect the viability of ACWR as a workload management tool. Furthermore, there is a lack of studies investigating the relationship between evidence-based wellness scores and the ACWR. The aim of this study was to investigate ACWR, training load, and wellness scores in male university field hockey players during an in-season phase of premier league hockey competition.

Some studies have highlighted that there may be methodological flaws with the ACWR method ^[8;9]^. However, the systematic review by Maupin et al.^[10]^ concluded that utilising the ACWR for external (e.g. total distance) and internal (e.g. heart rate) loads may be related to injury risk. Also, using exponentially weighted moving averages to calculate ACWR (as used in this study) may potentially result in a more sensitive measure. The review also stated that the methodological problems with the ACWR must be addressed before it can be confidently used to mitigate injury risk. A review by Gabbett ^11]^ noted that both coupled and uncoupled ACWRs have been associated with increased injury risk; however, the author stated that research comparing coupled and uncoupled ACWRs for detecting injury risk has yet to be conducted. Gabbett^[11]^ also suggested that rather than focusing solely on the ACWR, practitioners should account for known moderators of the workload-injury relationship (e.g. age, training and injury history, chronic workload, physical qualities), and the interpretation of internal and external load variables, together with well-being and physical readiness data. Therefore, the ACWR may be useful in workload management if analysed in conjunction with other factors that influence the workload-injury relationship, for example, interpreting a spike in ACWR in conjunction with the chronic workload and fitness level.^[11]^

This study did not address the debate of the relationship of the ACWR to injury risk but only investigated the relationship between the ACWR and wellness scores.

The objectives of the study were: (i) to describe the ACWRs during a premier league men’s field hockey season; (ii) to determine if there was an association between the ACWR and subjective, self-reported wellness scores; (iii) to determine if there was an association between training load and self-reported wellness scores of premier league male hockey players.

## Methods

### Participants

Fourteen male hockey players ranging between the ages of 19–24 years, and playing for the first team at the University of the Witwatersrand, Johannesburg, South Africa, participated in this study (mean ± SD; weight = 72.8 ± 8.0 kg, height = 174.7 ± 6.1 cm). The original sample group consisted of the entire men’s first team (twenty-three players); however, those who did not fill out the self-reported wellness and training questionnaires adequately (80% completion rate) were excluded from the study. The completion rate was calculated as the number of sessions reported by a player, divided by the total number of the team’s sessions over the study period. Players with completion rates of > 80% were included in the analysis of this study. Prior to data collection, the players were required to provide a signed, written informed consent form to partake in this study.

### Ethical considerations

Ethical clearance was obtained from the Human Ethics Committee of the University of the Witwatersrand (Protocol number: M190637). Anonymity of participants was achieved through the use of codes for each individual. Only the head researcher, supervisor and team coaches had access to the players’ identities. The raw data and results were password protected on a shared worksheet only accessible to the head researcher, supervisor, and team coaches.

### Study design

This study was a prospective, longitudinal, cohort, correlational study. The Google form included an RPE score for the session (sRPE) on a scale from 1 to 10 (10 being the maximal exertion and 1 being rest).^[12]^ The type of physical activity completed during the session (training, match, gym or other) and the duration of the session in minutes were also recorded. Athletes completed a wellness questionnaire which included five wellness scores (fatigue, sleep quality, general muscle soreness, stress level and mood) on a Likert scale from 1 to 5. Each player was asked to fill out the section on the Google form (sent via a link to their mobile phone) on training duration and sRPE at the end of every training/exercise session/match, as part of the team’s monitoring practices. The wellness scores were completed before bedtime every night. This is in line with other studies in which recovery questionnaires have been completed before bedtime.^[13]^ This study’s choice to complete the wellness questionnaire at bedtime was because the study’s questionnaires investigated the effect of an exercise session on exertion and wellness, over the past 24 hours’ exercise sessions and wellness perceptions.

### Quantifying the ACWR

sRPE-based training load was defined as the sRPE multiplied by the session’s duration in minutes (arbitrary units x minutes). The measurement of the ACWR has been validated as a workload management tool in previous research.^[11,12]^ Acute (one week) and chronic (four weeks) workloads were calculated using the exponentially weighted moving average (EWMA) model to describe the ACWRs of premier league male hockey players. This model places greater emphasis on the athlete’s most recent workload and a decreased weighting on older workload.^[14]^ The EWMA model places greater importance on the more recent workouts as they will have a greater effect on current training adaptations when compared to the older workouts. The ACWR was then calculated as the ratio of the acute workload calculation divided by the chronic workload calculation.

### Self-reported wellness

The general wellbeing of players was quantified by the use of a previously validated wellness questionnaire.^[15]^ Players filled in the questionnaire using a 1 to 5 Likert scale to rate their fatigue, sleep quality, general muscle soreness, stress level and mood (1 was rated as poor and 5 was rated as very good). A rating of 5 for fatigue or stress, for example, was equated to the least amount of fatigue or stress. The z-scores of the wellness scores were separately calculated and analysed to determine the degree of week-to-week changes in the athletes’ responses [z-score = (current rating – baseline rating)/standard deviation].

### Statistical analysis

Data were categorised into weekly blocks from Monday to Sunday. The dependant variables (ACWR and training load) were log-transformed before statistical analysis was undertaken using the mixed-effects model. The mixed-effects model was used to determine the percentage increase/decrease in relation to a unit increase in the independent variable (i.e. wellness scores). The relationship between log-transformed ACWR and wellness scores, as well as the relationship between log-transformed training load and wellness scores, was analysed using linear with mixed-effects regression. For an association between individual players’ training load and self-reported wellness scores, the multiple linear regression model was used. Data were analysed using STATA (v16) statistical software. A p-value less than 0.05 was considered significant.

## Results

### ACWR and training load

The ACWR of the players during the in-season showed a mean ACWR ranging from 0.67 to 1.89 and a mean (±SD) training load (AU) ranging from 489 ± 195 to 814 ± 303 (Table 1).

### ACWR and wellness scores

**Fig. 1:**
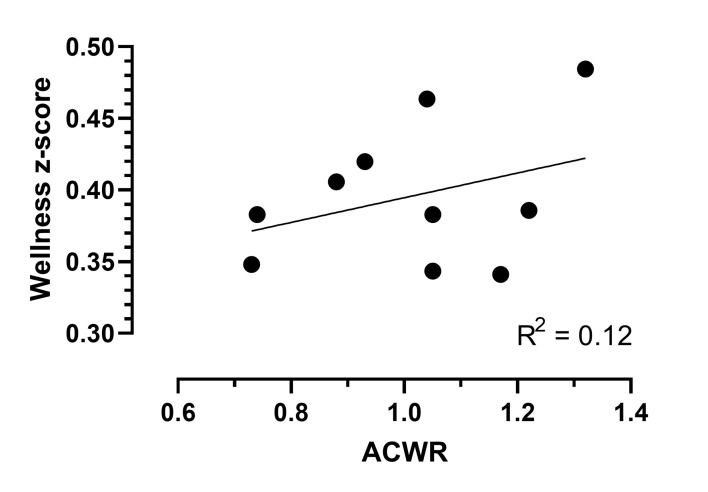
ACWR and wellness z-scores over a training cycle of 10 weeks. ACWR, acute:chronic workload ratio.

A linear mixed-effects regression model estimates describing the relationship between the log (ACWR) and each of the five wellness score parameters are shown in Table 2. ACWR was only associated with general muscle soreness (coefficient: −0.183, CI: −0.324, −0.043, p=0.01). A unit increase in general muscle soreness showed an 18% decrease in ACWR (Table 2). This indicated that muscle soreness improved with the players training less. Similarly, a unit increase in fatigue showed a 4.1% decrease in ACWR (coefficient: −0.041, CI: −0.182, 0.09, p=0.561), and a unit increase in sleep quality showed a 5.7% decrease in ACWR (coefficient: −0.057, CI: −0.185, 0.071, p=0.386). A unit increase in mood (i.e. better mood) showed a 4.9% increase in ACWR (coefficient: 0.049, CI: −0.89, 0.186, p=0.49) and a unit increase in the stress level (i.e. less stress) resulted in a 3.9% increase in ACWR (coefficient: 0.039, CI: −0.085, 0.162, p=0.54) (Table 2). An increase in weeks of training showed a reduction for ACWR ranging from 2.4%–2.7% every week, controlling for the different parameters (fatigue, sleep quality, general muscle soreness, stress, and mood) (Table 2). Fig. 1 provides a visual representation of the weekly training load fluctuations and the athletes’ summative wellness score responses. The wellness z-scores ranged from 0.30–0.48 and the ACWR had minimal to no influence on a cumulative wellness score, only explaining 12% of the variation (Fig. 1).

### Training load and wellness scores

This linear mixed-effects regression model estimates describing the relationship between the log (training load) and the five wellness score parameters are shown in Table 3. The training load was found to be associated with fatigue, sleep quality, general muscle soreness and mood. The wellness questionnaire was based on a Likert scale ranging from 1(feeling as bad as possible) to 5 (feeling as good as possible). The number 5 on the Linkert scale represented the positive end of the continuum. Therefore, a positive change for the five wellness scores would be indicated by a unit increase in the wellness score.

**Fig. 2:**
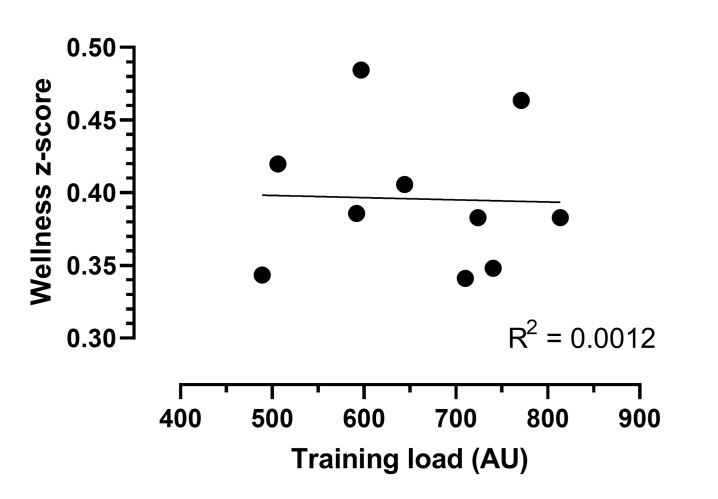
Training load and wellness z-scores over a training cycle of 10 weeks. Training load (AU) = sRPE x duration of session (in minutes).

A unit increase in fatigue (i.e. less fatigue present) showed a 22% decrease in the training load (coefficient: −0.215, CI: −0.349, −0.081, p=0.002). A unit increase in sleep quality (i.e. better sleep quality) showed a 12% decrease in the training load (coefficient: −0.12, CI: −0.234, 0.0001, p=0.05). A unit increase in general muscle soreness (i.e. less muscle soreness) showed a 20% decrease in the training load (coefficient: −0.201, CI: −0.340, −0.063, p=0.004). A unit increase in mood (i.e. better mood) showed a 15% decrease in the training load (coefficient: −0.151, CI: −0.283, −0.02, p=0.025), and a unit increase in the stress level (i.e. less stress) resulted in a 11% decrease in the training load (coefficient: −0.109, CI: −0.226, 0.008, p=0.068) (Table 3). A significant association was found between fatigue (p=0.002), sleep quality (p=0.05), general muscle soreness (p=0.004), mood (p=0.025) and the training load (Table 3). However, no association was found between the stress level and the training load. An increase in weeks of training showed a 2.0% − 2.5% reduction in the training load every week, controlling for each of the five different wellness scores (Table 3). Fig. 2 provides a visual representation of the weekly training load and the athletes’ summative wellness score responses. The training load had a minimal to no influence on a summative wellness score, which only explains 0.1% of the variation.

**Fig. 3:**
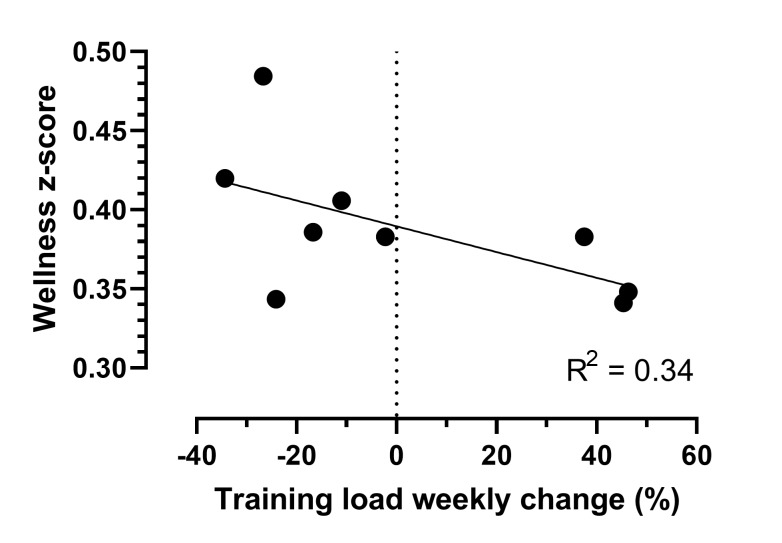
Weekly percentage changes in training load and wellness z-scores over the 10 week training cycle. Training load weekly percentage changes = [(current week training load – previous week training load)/ current week training load] x 100.

Fig. 3 depicts the weekly percentage changes in the training load, which ranged from a decrease of 34% to an increase of 46%. The training load’s weekly change had a weak influence on the summative wellness scores, equalling only 34% of these changes (Fig. 3).

Table 4 shows the results for the effects of the wellness scores on the training load for each individual player in the study. Generally, the training load was lighter with a “better” (higher) wellness score. Player 5 had a significant reduction of 20%, and 14% in his training load, with a decrease in fatigue and improved general muscle soreness, respectively. Player 9 had a significant reduction in his training load of 31% and 27% for a decrease in fatigue and improved sleep quality scores, respectively. Players 10 and 14 had significant reductions in their training loads, with a decrease in fatigue. Player 13 manifested significant reductions in the training load, with decreases in fatigue, improvement in sleep quality and better mood scores.

The average ACWR and wellness z-scores [z-score = (current score – average)/standard deviation] per week of all the players are represented in Fig.1. The wellness z-score uses the sum of all the individual wellness scores (i.e. fatigue, sleep quality, general muscle soreness and mood).

The average training load and wellness z-scores [z-score = (current score – average)/standard deviation] per week of all the players are represented in Fig. 2. The wellness z-score uses the sum of all the individual wellness scores (i.e. fatigue, sleep quality, general muscle soreness and mood).

The average percentage (%) changes in training load and wellness z-scores [z-score = (current score – average)/standard deviation] per week of all the players are represented in Fig. 3. The wellness z-score uses the sum of all the individual wellness scores (i.e. for fatigue, sleep quality, general muscle soreness and mood).

## Discussion

This study investigated the relationship between wellness scores (fatigue, sleep quality, general muscle soreness, stress level and mood) and the ACWR, as well as between the wellness scores and training load.

The average ACWR reported for all the players during the study period ranged from 0.67 to 1.89. There were no injuries reported during the study period. It should also be noted that the risk of injury may have been mitigated due to the injury prevention practice of adjusting the player training loads according to their wellness scores. It could also have been due to an optimal level of physical conditioning that had been developed in the pre-season.

The ACWR range in this study is larger than that reported in other studies. Research has proposed a ‘sweet spot’ range for the ACWR of 0.8 to 1.3, within which the relative risk of injury is thought to be minimised.^[10]^ In addition, an ACWR above 1.5 is considered the ‘danger zone’ for an increased risk of injury within 7–10 days.^[12]^ A study on professional soccer players found this ‘sweet spot’ to be between 1.00 and 1.25^[6]^, while another study showed it to be between 0.85 and 1.35 in elite rugby players^[5]^. A separate study investigating Gaelic football players found the ‘sweet spot’ to be between 1.35 and 1.5^[7]^.

A possible explanation for the reported greater range of the ACWR in this study could be due to the different nature of the sport type (field hockey) investigated, and has a wider range. Field hockey also utilises the rule of rolling substitutions during matches. These substitutions make it challenging for players to know the exact number of minutes realistically played during the game when reporting it for the calculation of the ACWR. Owing to the duration of the session being part of the ACWR calculation, the ACWR may have been skewed because the player did not report the exact duration of the session. However, this would have only applied to matches. The small sample size of the study could also have contributed to this finding.

This study found that the ACWR was only associated to one of the five subjective wellness score measurements i.e. general muscle soreness. Fatigue, sleep quality, mood and stress levels showed no association with the ACWR, yet general muscle soreness was significantly associated with ACWR (Table 2). This suggests that a player reporting more general muscle soreness (i.e. towards the Likert scale number of 1), may be related to the weekly increase in workload as measured by the ACWR. The training load was found to be significantly associated with more wellness measurements (i.e. fatigue, sleep quality, general muscle soreness and mood), when compared to the ACWR. Fatigue, sleep quality, general muscle soreness and mood were associated to the training load, but not to stress levels (Table 3). This may indicate that an increase in training load is associated with a player reporting increased fatigue levels, increased muscle soreness and which may affect sleep quality. The individual player responses confirmed this association between the training load and wellness scores by indicating that the training load was lighter with a “better” (higher) wellness score and that the players in our study generally had a homogeneous response to higher wellness scores (Table 4).

Team response analysis found no association with stress levels (Table 3*)*. This may be because these factors take effect when a player remains in a high stress or low mood level for a substantial time period. However, this was beyond the scope of this study.

The association of some of the wellness scores (fatigue, sleep quality, muscle soreness, mood and stress level) to the ACWR and the training load, show that subjective wellness scores could be a consideration when prescribing a training load to a field hockey player, as a means of monitoring this player’s response to the proposed training load. Furthermore, the differing associations between the two measures (ACWR and the training load) and wellness scores may indicate that the two measures could be measuring different aspects of a player’s workload.

Other studies have also investigated subjective measures (wellness scores) in the context of training, but these measures were collected before the athletes participated in their training session or match. ^[15–17]^ These other studies covered Australian football or American college football players. They found that pre-training subjective measures may provide useful information to coaches, such as a player’s level of internal load, as well as potentially indicating a player’s readiness to train. In this study; however, wellness scores (and sRPEs) were reported after a training session or match (before bedtime).

Research has also found the sRPE-based training load to be a good indicator of internal load in soccer ^[18]^. It was theorised to be a good indicator of a player’s capacity before a training session and a key determinant response to training in American college football.^[17]^ Govus et al.^[17]^ found a 1-unit increase in muscle soreness (meaning the player was feeling less sore) which was associated with a 4.4% decrease in the sRPE-based training load in American college football. This study found the same unit increase in muscle soreness to be associated with a 28% decrease in the sRPE-based training load. A difference in risk factors between the two sports and individual athletes, likely due to varied physiological capacities and physical demands of each sport, could explain the disparity between the two results.

Ihsan et al.^[19]^ investigated male field hockey players and examined the association between some of the wellness measures (fatigue, muscle soreness, mood state and sleep quality) and match running performance over the course of an international field hockey tournament. The results were normalised for playing time, or post-match RPE, or for both playing time and post-match RPE. Ihsan et al.’s study found that changes in a player’s running performance were better associated to changes in wellness scores when running performance was normalised for both playing time as well as post-match RPE. Such findings support evidence for the use of wellness measures as pre-match tools to assist in managing internal load of hockey players during a tournament.^[19]^ This study however, used post-training/match data collection and could thus not compare research findings.

### Limitations

Hockey is a sport which utilises rolling substitutions throughout the match. Therefore, this may skew the subjective wellness data of players as they are often unsure of how many minutes they have realistically spent playing. This results in possibly skewed ACWR measurements and could make it challenging for the workload to be accurately quantified using self-reported measures. Even though the entire team (n = 23 men) were originally included in the study, only fourteen were analysed in the final report. This lack of compliance from players is a constant battle for sports practitioners and limits the accuracy, as well as the generalisability, of the results due to the smaller sample group. Collaborating with players so that they understand the value of filling out such questionnaires for their performance enhancement is a suggestion to help improve the compliance of players. A longer period of observation would have been optimal to monitor the players’ workloads, including through their pre-season training. Pre-season training is notoriously rigorous and intense as the players are required to reach peak physical conditions in time for the season and then to maintain that peak condition throughout the entire season. The process of the players reaching this level of optimal fitness may add a new level of importance to a study such as this one.

An additional limitation of this study was that it did not control for recall biases with the wellness questionnaire that was filled out at bedtime, especially the previous night’s sleep quality. The wellness scores were filled out at some stage during the night of the training/match session to ensure that it is in agreement with the completion of the ACWR recording that was done post exercise sessions. This study, by investigating, the relationship between ACWR and wellness scores, tried to keep the timing of the reporting of these measures as close as possible. Research using the Total Quality Recovery Scale has shown that reporting measures post 24 hours is still useful. ^[13,20]^

### Study strengths and future directions

The strengths of this study include:

- The use of the EWMA to calculate the ACWR - not rolling averages, which is statistically a better method. It is also a more sensitive method for identifying changes in the chronic load and adaptation to training load.^[14]^
- With a small sample of players which were included in this study, using the linear mixed-effects method to assess the association between performance indicators (ACWR and training load) and wellness score parameters gives strength to the observed results. The mixed-effects methods incorporated both the fixed and random effects and is suitable for follow-up studies with dependent participant measurements.
- The study included training sessions and competition in our analysis, which gave a more complete picture of loading, as opposed to analysing only one aspect of workload (e.g. matches).

Further studies need to be conducted on the association between the ACWR and injury risk in outdoor field hockey across a longer duration (ideally including the pre-season training period), in order to formulate a more accurate estimate of the ACWR’s ‘sweet spot’. It is suggested that the subjective measures, such as internal load used in this study, are investigated with a larger sample size and in conjunction with external load measures (such as, distance travelled, running speed etc. measured via GPS systems) in field hockey. This could allow for more accurate monitoring of a hockey player’s response to the training load and may be used as a predictor for readiness to train.

## Conclusion

This study monitored hockey players over the course of a season and considered wellness scores in relation to ACWRs and the sRPE-based training load. Associations between certain wellness scores and the ACWR (i.e. with general muscle soreness), and the sRPE-based training load (i.e. with fatigue, sleep quality, general muscle soreness and mood) were found. Therefore, there may be some merit in the use of subjective, individual wellness measures as workload management tools in field hockey. However, when a summative wellness score is used (wellness z-score), ACWR and the training load seem to have minimal to no influence on any wellness scores. Training load change per week seems to have a weak influence on all wellness scores.

## Figures and Tables

**Table 1:** ACWR, training load and wellness scores over the 10-week period of the study (n=14)

Week	ACWR	Training load (AU)*	Fatigue	Sleep quality	General muscle soreness	Stress level	Mood
1	1.89 ± 0.97	771 ± 333	2.9 ± 0.6	2.9 ± 0.6	2.7 ± 0.6	2.9 ± 1.0	3.6 ± 0.4
2	0.86 ± 0.53	506 ± 294	2.8 ± 0.9	2.9 ± 0.9	3.1 ± 0.7	2.5 ± 0.7	3.4 ± 0.4
3	0.68 ± 0.62	741 ± 314	2.6 ± 0.8	3.4 ± 1.1	2.8 ± 0.9	2.4 ± 0.8	3.2 ± 0.8
4	1.01 ± 0.56	724 ± 372	2.7 ± 0.7	3.0 ± 0.8	2.7 ± 0.4	2.6 ± 0.6	3.3 ± 0.6
5	0.99 ± 0.61	644 ± 240	2.6 ± 0.6	2.8 ± 0.9	2.7 ± 0.8	2.7 ± 0.8	3.3 ± 1.0
6	0.67 ± 0.46	489 ± 195	2.6 ± 0.8	3.3 ± 0.9	2.5 ± 0.7	2.8 ± 0.9	3.2 ± 0.8
7	1.01 ± 0.51	710 ± 378	2.8 ± 0.7	3.2 ± 0.9	3.1 ± 0.6	2.5 ± 0.6	2.9 ± 0.9
8	1.14 ± 0.40	592 ± 376	2.7 ± 0.5	2.9 ± 0.8	2.7 ± 0.5	2.4 ± 0.6	3.0 ± 0.7
9	1.12 ± 0.36	814 ± 303	2.8 ± 0.3	3.0 ± 0.7	2.8 ± 0.6	3.0 ± 0.7	3.7 ± 0.7
10	0.94 ± 0.38	597 ± 191	3.0 ± 0.0	2.9 ± 0.7	2.6 ± 0.4	3.4 ± 0.6	3.4 ± 0.3

Data are expressed as mean ± SD.

* Training load = time of session x sRPE.

ACWR, acute:chronic workload ratio; sRPE, session rating of perceived exertion.

**Table 2:** Linear mixed-model parameter estimated and 95% CIs for the relationship between ACWR and subjective wellness scores (n=14)

Fixed effect	Model 1	Model 2	Model 3	Model 4	Model 5	p
**Fatigue**	−0.041 [−0.182, 0.09]					0.56
**Sleep quality**		−0.057 [−0.185,0.071]				0.39
**General muscle soreness**			−0.183 [−0.324,−0.043]			0.01*
**Stress level**				0.039 [−0.085, 0.162]		0.54
**Mood**					0.049 [−0.089, 0.186]	0.49
**Week**	−0.026 [−0.058,0.005]	−0.025 [−0.057, 0.007]	−0.027 [−0.058, 0.003]	−0.026 [−0.058, 0.005]	−0.024 [−0.057, 0.007]	

Data are expressed as coefficient estimates [95% confidence interval]. Week indicates the change of ACWR from one week to the next.

* indicates p<0.05.

ACWR, acute:chronic workload ratio.

**Table 3:** Linear mixed-model parameter estimated and 95% CIs for the relationship between training load and subjective wellness scores (n=14)

Fixed effect	Model 1	Model 2	Model 3	Model 4	Model 5	p
**Fatigue**	−0.215 [−0.349, −0.081]					0.00*
**Sleep quality**		−0.120 [−0.234, 0.0001]				0.05*
**General muscle soreness**			−0.201 [−0.340, −0.063]			0.00*
**Stress level**				−0.109 [−0.226, 0.008]		0.07
**Mood**					−0.151 [−0.283, −0.02]	0.03*
**Week**	−0.021 [−0.060, 0.0167]	−0.020 [−0.056, 0.021]	−0.021 [−0.060, 0.020]	−0.020 [−0.057, 0.018]	−0.025 [−0.06, 0.012]	

Data are expressed as coefficient estimates [95% confidence interval]. Week indicates the change of training load from one week to the next.

* indicates p<0.05.

Training load = time of session x sRPE.

**Table 4:** Regression coefficients (p-values) for wellness scores on log (training load) adjusting for the duration of activity (training/play/gym) and type of session

Player	Fatigue	Sleep quality	General muscle soreness	Stress level	Mood
1	−0.03 (0.44)	−0.001 (0.98)	−0.03 (0.30)	0.05 (0.34)	0.04 (0.45)
2	−0.08 (0.30)	−0.04 (0.54)	−0.06 (0.36)	0.02 (0.78)	0.07 (0.52)
3	−0.02 (0.79)	0.06 (0.24)	−0.03 (0.54)	0.04 (0.47)	0.01 (0.86)
4	−0.23 (0.09)	0.18 (0.45)	−0.10 (0.33)	−0.05 (0.68)	Omitted
5	−0.20 (0.01*)	−0.04 (0.46)	−0.14 (0.02*)	0.02 (0.69)	−0.06 (0.33)
6	−0.20 (0.12)	−0.06 (0.37)	−0.12 (0.15)	0.003 (0.97)	−0.19 (0.12)
7	Omitted	−0.04 (0.84)	0.03 (0.82)	−0.16 (0.31)	Omitted
8	0.05 (0.16)	0.03 (0.41)	0.02 (0.74)	0.02 (0.61)	0.05 (0.16)
9	−0.31 (0.01*)	−0.27 (0.01*)	−0.13 (0.14)	−0.12 (0.18)	−0.23 (0.11)
10	−0.15 (0.04*)	−0.10 (0.13)	−0.04 (0.50)	0.11 (0.31)	0.01 (0.93)
11	−0.32 (0.20)	−0.29 (0.23)	−0.34 (0.13)	−0.12 (0.48)	−0.23 (0.13)
12	−0.20 (0.15)	−0.09 (0.38)	−0.13 (0.30)	−0.001 (0.99)	0.20 (0.09)
13	−0.20 (0.04*)	−0.29 (0.03*)	−0.07 (0.48)	0.16 (0.10)	−0.33 (0.01*)
14	−0.07 (0.04*)	−0.01 (0.85)	−0.021 (0.74)	−0.03 (0.38)	−0.05 (0.27)

Data are expressed as regression coefficients (p-values).

* indicates p<0.05.

Omitted indicates no variation on the wellness scores across all measured instances.
